# New insights into *Arabidopsis* transcriptome complexity revealed by direct sequencing of native RNAs

**DOI:** 10.1093/nar/gkaa588

**Published:** 2020-07-11

**Authors:** Shoudong Zhang, Runsheng Li, Li Zhang, Shengjie Chen, Min Xie, Liu Yang, Yiji Xia, Christine H Foyer, Zhongying Zhao, Hon-Ming Lam

**Affiliations:** School of Life Sciences and Center for Soybean Research of the State Key Laboratory of Agrobiotechnology, The Chinese University of Hong Kong, Shatin, Hong Kong Special Administrative Region; Department of Biology, Faculty of Science, Hong Kong Baptist University, Kowloon Tong, Hong Kong Special Administrative Region; Department of Infectious Diseases and Public Health, Jockey Club College of Veterinary Medicine and Life Sciences, City University of Hong Kong, Kowloon, Hong Kong; School of Life Sciences and Center for Soybean Research of the State Key Laboratory of Agrobiotechnology, The Chinese University of Hong Kong, Shatin, Hong Kong Special Administrative Region; School of Life Sciences and Center for Soybean Research of the State Key Laboratory of Agrobiotechnology, The Chinese University of Hong Kong, Shatin, Hong Kong Special Administrative Region; School of Life Sciences and Center for Soybean Research of the State Key Laboratory of Agrobiotechnology, The Chinese University of Hong Kong, Shatin, Hong Kong Special Administrative Region; School of Life Sciences and Center for Soybean Research of the State Key Laboratory of Agrobiotechnology, The Chinese University of Hong Kong, Shatin, Hong Kong Special Administrative Region; Department of Biology, Faculty of Science, Hong Kong Baptist University, Kowloon Tong, Hong Kong Special Administrative Region; The State Key Laboratory of Agrobiotechnology, The Chinese University of Hong Kong, Shatin, Hong Kong Special Administrative Region; School of Biosciences College of Life and Environmental Sciences, University of Birmingham, Edgbaston, B15 2TT, UK; Department of Biology, Faculty of Science, Hong Kong Baptist University, Kowloon Tong, Hong Kong Special Administrative Region; School of Life Sciences and Center for Soybean Research of the State Key Laboratory of Agrobiotechnology, The Chinese University of Hong Kong, Shatin, Hong Kong Special Administrative Region

## Abstract

*Arabidopsis thaliana* transcriptomes have been extensively studied and characterized under different conditions. However, most of the current ‘RNA-sequencing’ technologies produce a relatively short read length and demand a reverse-transcription step, preventing effective characterization of transcriptome complexity. Here, we performed Direct RNA Sequencing (DRS) using the latest Oxford Nanopore Technology (ONT) with exceptional read length. We demonstrate that the complexity of the *A. thaliana* transcriptomes has been substantially under-estimated. The ONT direct RNA sequencing identified novel transcript isoforms at both the vegetative (14-day old seedlings, stage 1.04) and reproductive stages (stage 6.00–6.10) of development. Using in-house software called TrackCluster, we determined alternative transcription initiation (ATI), alternative polyadenylation (APA), alternative splicing (AS), and fusion transcripts. More than 38 500 novel transcript isoforms were identified, including six categories of fusion-transcripts that may result from differential RNA processing mechanisms. Aided by the Tombo algorithm, we found an enrichment of m5C modifications in the mobile mRNAs, consistent with a recent finding that m5C modification in mRNAs is crucial for their long-distance movement. In summary, ONT DRS offers an advantage in the identification and functional characterization of novel RNA isoforms and RNA base modifications, significantly improving annotation of the *A. thaliana* genome.

## INTRODUCTION

Transcriptome analysis provides a powerful approach for identification and quantification of RNA transcripts and their processed forms (1). Previous studies with next generation sequencing (NGS) and PacBio long-read sequencing demand conversion of RNA into cDNA, which may fail to identify mRNA complexity. Oxford Nanopore Technology (ONT) is capable of directly sequencing native full-length RNA molecules, whereas the NGS-based platforms can only produce much shorter reads (35–700 bp) (2,3). Therefore, the capability of NGS systems to characterize the complexity of the mRNA transcriptome, particularly base modifications, is compromised. Relatively few transcriptome analyses have been conducted using ONT Direct RNA Sequencing (DRS) in *Arabidopsis* (4). A comprehensive catalogue of transcript isoforms using the ONT long reads has not been made to date because of limitations in the protocols used for library preparation. Crucially, the RNA G-Quadruplex (rG4) structure (5) has not been considered when preparing libraries for ONT DRS. This problem has been further complicated by the lack of a customized algorithm for the systematic discovery of novel isoforms.

In the present study, we demonstrate the power of ONT DRS for the identification of processed mRNA isoforms and RNA modifications. By sequencing the full-length native mRNAs at two different developmental stages in *A. thaliana*, we identified more than 38 500 novel transcript isoforms that are currently missing from the Araport11 database (6). We experimentally-validated a subset of the novel isoforms. Cytosine methylation of mRNAs is regarded as a crucial marker for mobile mRNAs. The disruption of m5C modifications in the gene encoding *Arabidopsis*TRANSLATIONALLY CONTROLLED TUMOR PROTEIN (TCTP1) abolished long-distance movement (7). Using the ONT DRS, we also identified putative m5C RNA modifications and shown that these modifications exhibit a positive correlation with the long-distance movement of mobile mRNAs.

## MATERIALS AND METHODS

### Plant materials and growth conditions

*Arabidopsis thaliana* col-0 seeds were surface sterilized with 50% commercial bleach and 0.01% Triton X-100 for 10 min, then washed five times with sterilized H_2_O. The sterilized seeds were sown on $\frac{1}{2}$ MS plate with 1% sucrose and 0.6% Agar (sigma, cat#: A1296). Stratification at 4°C for 48 h, then placed in a growth chamber with 16 h light/8 h dark for 14 days. The 14-day seedlings were harvested (three biological replicates, each with 1 g, one sample was used for an ONT DRS pilot run, but these data are not included in the present datasets) for RNA extraction. Some seedlings were transplanted into soil for floral buds, the soil-based *Arabidopsis* seedlings were also treated with photoperiod of 16 h light/8 h dark. After another three-week growing, floral buds from plants equals to principal growth stage 6.0 to 6.1 were harvested, and they were immediately immersed in a foil boat full of liquid nitrogen in a relative larger liquid nitrogen container. Two harvested biological replicates were frozen at −80°C freezer till RNA isolation.

### RNA extraction and Nanopore direct RNA sequencing library preparation

Total RNAs from *Arabidopsis* seedlings and floral buds were isolated with Trizol according to manufacturer's instruction. The extracted total RNAs were then treated with DNase I (NEB CAT#:M0303). The treated total RNAs were extracted with acidic phenol (125:24:1, pH 4.5, ThermoFisher cat#: AM9720) and precipitated with 2.5 M LiCl for two replicates from seedlings and two from floral buds. The purified RNAs were subjected to mRNA isolation with Dynabeads mRNA purification kit (ThermoFisher cat#: 61006), around 0.9 ug mRNAs for each library were used with Nanopore direct RNA sequencing kit (SQK-RNA002). The prepared libraries were loaded onto R9.4.1 flowcells, and sequencing was performed in MinION sequencer. Each library was run for 48 h.

### RT-PCR and Chop-RT-PCR

For RT-PCR, 1 ug total RNAs were used for cDNA synthesis with TransScript one-step gDNA removal and cDNA synthesis supermix kit (Catalog Number:AT311-02) as manual suggested. The RT reaction solution were diluted with five times of ddH_2_O, and 1 ul from the diluted RT solution was used for each PCR reaction in a scaled 10 ul reaction system with Phusion DNA polymerase (Catalog Number: F530S), For chop-RT-PCR, the amplified RT-PCR were digested with marked enzymes in Supplementary Figure S3 for 2 h. The RT-PCR and chop-RT-PCR products were separated with 1.5–2.0 % Agarose gel.

### Poly(A) length estimation

The poly(A) lengths of each read were calculated using Nanopolish (version 0.11.1) with ‘polya-merged’ branch (8). The raw ion signal from the 3′ unaligned ends of reads were extracted to estimate the length of poly-(A) tail, which was deduced by the dwell time of the signal.

### Alternative polyadenylation (APA) analysis

The polyadenylation (poly(A) sites for each DRS read were recorded. Sites with >5-read support were selected for inclusion in the APA list. This list was compared with the APA lists obtained from Araport11, Wu *et al.* (9) and Sherstney *et al.* (10), respectively. The poly(A) sites with 5 nt shifts between different datasets were identified as the same modification. For the APA motif analysis, a 50-nt region upstream of poly(A) sites was scanned for all possible hexamer sequences so that the top 50 overrepresented motifs could be identified. The overrepresented motifs were then scanned against the sequences of 14–24 nt (19 ± 5 nt) upstream of a poly(A) sites to obtain data on the occurrence of the motifs within these regions using SignalSleuth2 (11).

### DEG and DEI calculation

An isoform was defined as differentially expressed isoform (DEI) between two tissues when the change of its relative abundance (percentage of read count) out of all the transcripts within the same locus is greater than 10%. A gene was defined as differentially expressed gene (DEG) between tissues when the fold change of its abundance of combined transcripts (read count per million) was greater than two and false discovery rate (FDR) was <0.05. The FDR for the DEGs was calculated in R package edgeR (version 3.30) (12), with ‘quantile normalization’ method for normalization.

### External data source

Several published datasets were used for comparison of the results obtained by Nanopore DRS and literature data. We used two Illumina short-read RNA-seq datasets for *A. thaliana* that include 14-day old seedlings (GSE104483) (13) and 8–10-day floral buds (GSE139917) (14). Two sets of Nanopore DRS data for 14-day-old wild-type *A. thaliana* seedlings (4) were also included. The Sequence Read Archive (SRA) accession number for all the sequencing data used is listed in Supplementary Table S5.

### Base calling and Nanopore long read analysis

The reads were re-base-called with the Guppy basecaller (version 3.1.5) (15), with high accurate model. The resulting sequences were separately mapped against the Araport11 transcriptome with parameters ‘-ax map-ont’ and against the TAIR10 genome with parameters ‘-ax splice -k 14 -G 100000’ using Minimap2 (16). The resulting ‘SAM’ files were sorted and indexed with ‘SAMtools’ (v2.1) (17) by sequence coordinate. For visualization on UCSC genome browser, the read mapping results were converted to bigGenePred format (https://genome.ucsc.edu/goldenpath/help/examples/bigGenePred.as) using a custom script embedded in the TrackCluster package (https://www.github.com/runsheng/trackcluster/) (18). The novel isoform calling, and isoform quantification was done by TrackCluster package, with default parameters. The TrackCluster related code can be found in Supplementary Data1.

### RNA base modification analysis with m5C model and *de novo* model

Modification of the RNA sequences were identified with Tombo package version 1.5 (19). The models of ‘5mC’ and ‘*de novo*’ were implemented separately to detect possible modification in each read. The score on each site indicated the fraction of a possible modification on a given site. For plotting the modification coverage along gene body and UTRs, the modification coverage was normalized for each isoform using ‘w0’ method with a bin size of five nucleotides (20). Only the isoforms with both 5′ and 3′ UTR longer than 50 nucleotides were used in the calculation.

## RESULTS

### ONT DRS enables the sequencing of full-length transcripts

**Figure 1.**
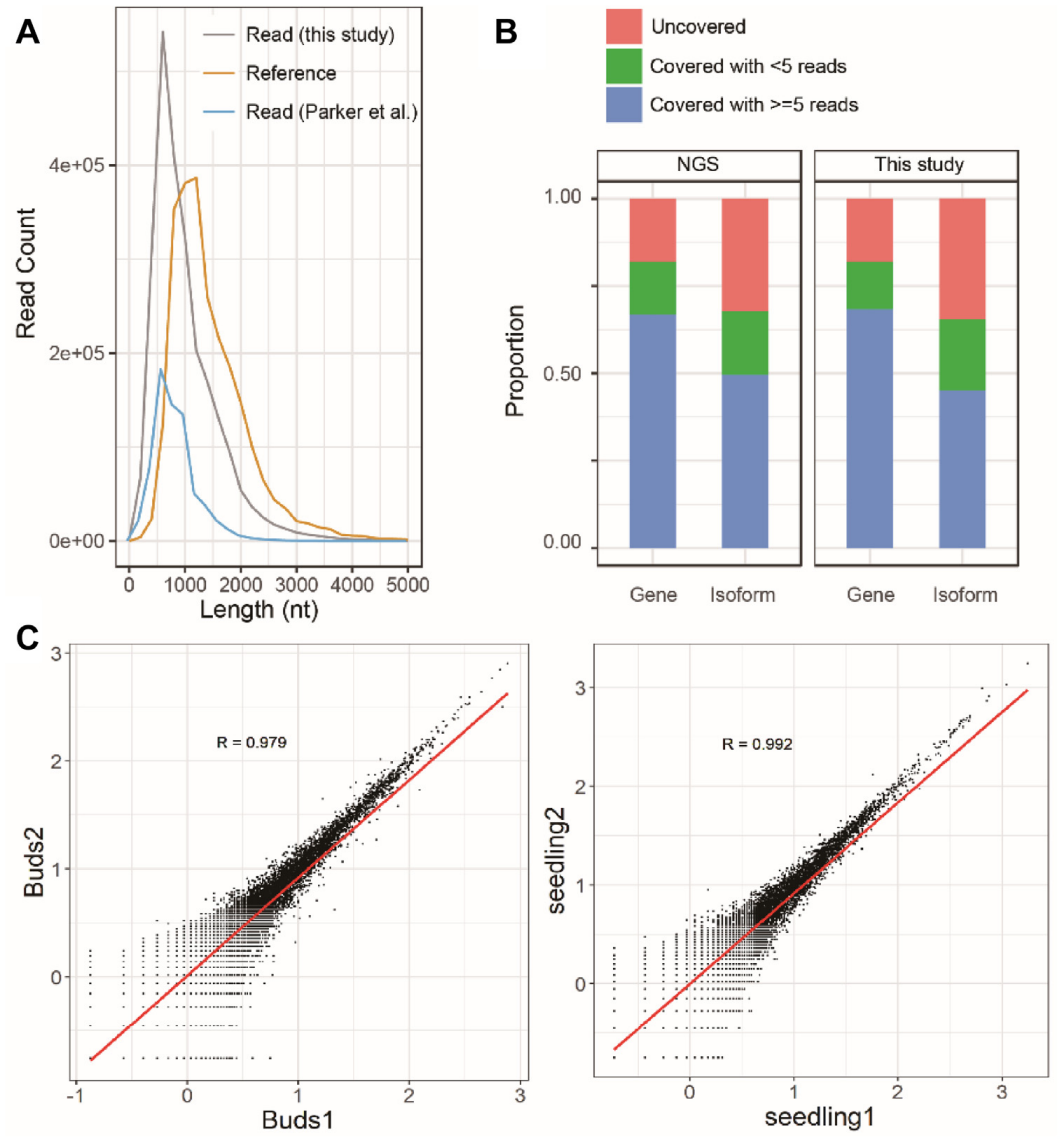
Statistical analysis of Nanopore reads and coverage as well as replicate reproducibility. (**A**) Read length distribution. Grey curve: the length distributions of Nanopore read; yellow curve: reference according to the existing longest isoforms of the gene were counted; blue curve: the length distributions of Nanopore reads from two of the seedling samples with 5′ adaptor from the publication of Parker *et al.* (4). (**B**) The proportion of the existing isoforms or genes in the Araport11 reference covered by all our long reads (right) or by the same amount of NGS reads (left). Red: not discovered genes or isoforms; green: genes or isoforms discovered by Nanopore read, but with <5 reads support; blue: discovered genes or isoforms with >5 reads support. (**C**) Correlation between two replicates of sequenced libraries. Shown are the biological replicates from floral buds (left) and seedlings (right).

We used ONT DRS to analyse the transcriptomes of 14-day-old *A. thaliana* seedlings grown on plates to developmental growth stage 1.04 (hereafter referred to as seedlings) (21) and unopened floral buds (developmental growth stage 6.0–6.1; hereafter referred to as floral buds) (21). Sequencing libraries prepared using purified polyadenylated RNAs were loaded onto R9.4.1 flow-cells and sequenced in a portable MinION sequencer. We generated approximately 3.2 million of long reads in total for all four libraries (two for floral buds, two for seedlings) with an average read length of 900–1000nt for each library (Table 1). The read length distribution approached the theoretical length distribution of *A. thaliana* mRNAs (Figure 1A), indicating a relatively high integrity of the sequenced RNAs. Using the base caller Guppy (Version 3.1.5) (15), the overall read accuracy for our samples was ∼93% (Table 1). This greatly improves the reliability of the AS analysis. Over 93.9% Nanopore reads were successfully mapped to the *A. thaliana* reference genome using Minimap2 through ‘split-read’ alignment (16). This procedure implements a ‘concave gap cost’ for long insertions and deletions to accommodate intron skipping. The substantially elevated mapping rate (compared to previous reads generated using the old base caller, e.g. Albacore) achieved over an extended genomic interval is extremely useful for the identification of novel splicing isoforms.

**Table 1.** Summary of Nanopore native RNA read statistics

Stage	Seedling1	Seedling2	Buds1	Buds2
Read number^a^	707 220	736 859	982 453	780 408
Average length	1003	983	1000	924
Median length	851	828	820	764
N50 length	1188	1143	1214	1141
Max read length	24 734	20 411	57 484	24 080
Average read quality	12	12	12	12
Alignment % identity^b^	93%	93%	92%	92%

^a^The Read number is the raw read number, excluding the ENO2 internal control.

^b^Reference of cDNA and genome were fetched from Araport11, containing 48 359 protein coding isoforms in total.

**Figure 2.**
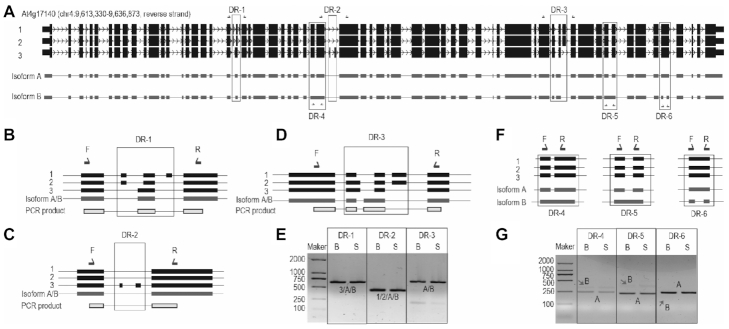
Correction of existing misannotated transcripts of At4g17140 by Nanopore long reads. (**A**) Diagram showing the difference between three reference isoforms (1–3, black) and our novel transcripts/isoforms (A and B, gray). Six different regions (DRs) are highlighted with box, and the primers used to amplify this region are shown near the DRs. DR 1–3 are the difference between reference isoforms and both the novel isoform A and B; while the DR 3–6 are the difference between isoform B and isoform A. (**B–D**). A magnified view of DR 1–3 shown in ‘A’. Shown on the top are the primers used for PCR validation and the mapping result of Sanger sequencing. (**E**). RT-PCR results for the confirmation of DR 1–3. Only one major band for each PCR can be found, and the isoforms supported by the Sanger sequencing results for each PCR are shown under the band. (**F**). A magnified view of DR 4–6 shown in ‘A’. (**G**). RT-PCR results for the confirmation of difference 4–6. The weaker band for the expected product of isoform B are indicated by red arrows. B, floral buds; S, seedlings; maker, the DL2000 DNA ladder. DR, different region.

When mapping the long reads to the existing annotated exons and UTRs in Araport11, about 82% (22 669 out of 27 655) of the protein-coding genes and 65% (31 658 out of 48 359) of the existing transcript isoforms were reproducibly recovered in two stages with two biological replicates (Figure 1B and C). The ONT DRS results are similar to those obtained by Illumina analysis of *Arabidopsis* seedlings (13) and floral buds at developmental stages 8–10 (14) (Figure 1B, left panel). The differential mapping ratios of ONT DRS reads against Araport11 genome (larger than 93.9%) and against the Araport11 transcriptome (∼82%) suggest that some of the ONT DRS reads may represent novel transcripts that are currently not annotated in the Araport11 database. One example is the super-long reads (>13 000 nt) mapped to the gene At4g17140. These reads were not included in our novel transcript isoform list because the copy number of the reads is smaller than five, the requirement for defining a novel isoform used in this study. Using the improved protocol (see Materials and Methods), we were able to generate three copies of reads that cover full-length isoform A and one copy that covers isoform B of the gene At4g17140, respectively. The Nanopore identified isoforms are different from all Araport11 annotated isoforms in three different regions (Figure 2A). The Nanopore reads in ‘Different-region (DR)-1’ have the same splicing patterns as the Araport11 annotated At4g17140.3 form but differ from At4g17140.1 and At4g17140.2 (Figure 2B). Moreover, the Nanopore reads in ‘DR-2’ have identical sequences to At4g17140.1 and At4g17140.2 but they differ from At4g17140.3 (Figure 2C). The ‘DR-3′, category has no reads to support any of the Araport11 annotated transcript isoforms. In DR-3, we found that the Nanopore reads contain an extended exon for most parts of intron 52 of At4g17140.3 because of the selection of an alternative 3′ acceptor site (Figure 2D). To verify the Nanopore results, we amplified the cDNAs in the relevant regions by RT-PCR with gene-specific primers (Figure 2E) followed by Sanger sequencing (Supplementary Data2). The results of this analysis confirmed that the transcripts derived from the Nanopore reads in all cases (Figure 2E). These findings demonstrate the power of ONT direct RNA sequencing for the identification of complex isoforms.

In addition to obtaining the correct combination of exons, we also detected a new full-length isoform of At4g17140 (isoform B; Figure 2A, F–G), which is different from isoform A at several regions including exon 60 (DR-6, Figure 2A, F), exons 56–57 (DR-5, Figure 2A, F), and exons 30–31 (DR-4, Figure 2A, F) counted using Araport11 annotated At4g17140.3. These differences were confirmed by gene-specific RT-PCR (Figure 2G).

### Novel isoforms identified by Nanopore long reads

**Figure 3.**
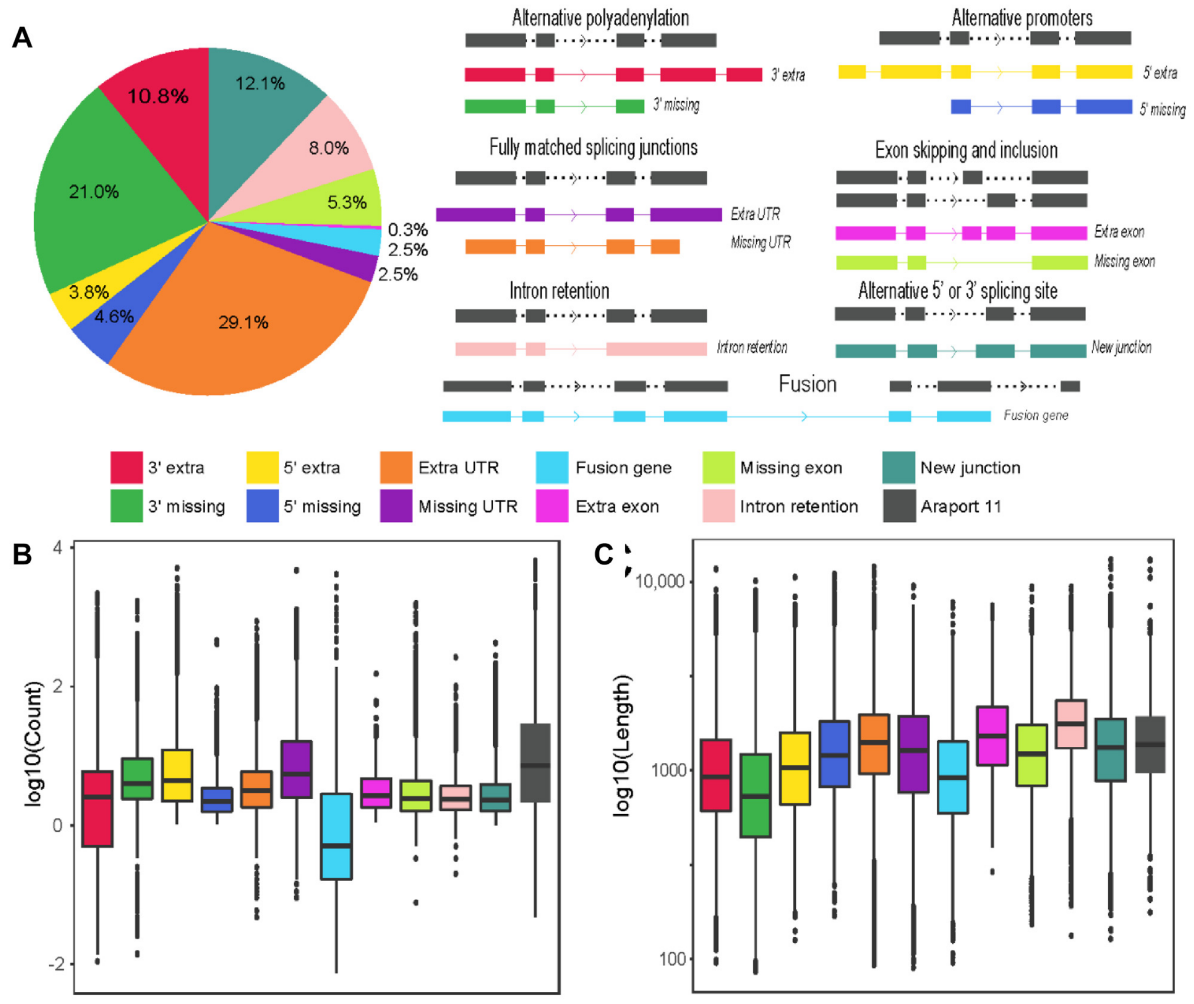
TrackCluster identified transcript isoforms and the distributions of read count and length for each category of isoforms. (**A**) Schematics of each category of novel isoform identified with Nanopore reads and their distribution. (**B**) Abundance of Nanopore reads mapped to various categories of isoform. (**C**) Distribution of read length for various categories of isoform in the study.

Using the customized pipeline called TrackCluster that was designed to identify and quantify novel transcript isoforms (18), we identified >38 500 novel transcript isoforms with the support of at least 5 reads (Supplemental Table S1). These isoforms represent a significant addition to the existing *A. thaliana* transcriptome. They can be classified into 11 categories. Four of these categories involve alternative use of promoters or polyadenylation sites, i.e. bearing extra or missing exon(s) at the 5′ or 3′ end (5′ extra, 3′ extra, 5′ missing and 3′ missing). Two categories involve UTR extensions or truncations at the 5′ or 3′ end, in which all the newly identified intron-exon boundaries match with those of an existing isoform except the first or the last exon (extra UTR and missing UTR). Two categories involve new combinations of exons within the gene body, including extra or missing exon(s) (extra exon and missing exon). One category involves intron retention, one category involves alternative splicing sites other than existing ones (new junctions), and the last category involves fusion of two separate isoforms from adjacent genes into a single isoform (fusion genes) (Figure 3A). Most of the newly discovered isoforms generally have a lower read coverage than the annotated transcripts in Araport11 (Figure 3B), suggesting that they are minor transcripts. As predicted, novel transcripts with extra UTRs, extra exons or intron retention have a relatively longer average length than the transcripts annotated in Araport11, while other categories of ONT transcript isoforms have a relatively shorter transcript length (Figure 3C).

The length of poly(A) tails is of physiological relevance because it regulates mRNA export, stability and translation over developmental stages or under different environmental conditions (22). Since the Nanopore direct RNA sequencing outputs ionic current profiles for every 5–6 nucleotides, when poly(A) goes through the protein Nanopores, the signals may exhibit no changes. However, the software Nanopolish (https://nanopolish.readthedocs.io/en/latest/quickstart_polya.html) is able to use the dwelling time to estimate the number of A’s in the poly(A) tails (8). Using this strategy, we analysed poly(A) length of mRNAs in seedlings and floral buds. Our results indicate that the average poly(A) size in floral bud mRNAs is slightly larger than that in seedling mRNAs. The majority of poly(A) lengths in both tissue types was <100nt. This is slightly longer than the recently reported poly(A) length obtained in *Arabidopsis* seedlings (4) (Supplementary Figure S1). Mature mRNAs from ribosomal protein genes and other house-keeping protein genes tend to have shorter poly(A) tails (23). This finding agrees with the recent observation that longer poly(A) tend to have incomplete processing of pre-mRNAs (e.g. intron retention reads in Nanopore direct RNA sequencing) (8).

3′UTR length is generally determined by APA sites. Recent studies have shown that 3′UTR length may depend on cellular conditions (24). APA produce different 3′UTR lengths that potentially regulate the functions, stability, localization, and translation efficiency of target RNAs (25). We employed ONT direct RNA sequencing to predict APA sites using a minimum of 5 reads. The resulting APA sites were compared with those documented in Araport11 and those obtained by PAT sequencing (9). Our ONT-based results show a higher similarity to those obtained by PAT-sequencing than to those documented in Araport11. Over 50% APA sites identified in this study were also detected by PAT-sequencing. APA sites produced with both ONT and PAT-seq methods demonstrate a modest overlap with those documented in Araport11 as shown in Supplementary Figure S2. Therefore, APA sites have been severely underestimated in the Araport11, which also was confirmed by Helico DRS (10). Both ONT direct RNA-seq and PAT-Sequencing revealed more APA sites than those annotated in Araport11 (Supplementary Figure S2A). However, our data show a higher consistency with those produced by Helico DRS than those obtained by PAT-Sequencing (Supplementary Figure S2B) (10). Searching for putative motifs used as polyadenylation signals with 109,880 APA sites identified by ONT, we found that the top 10 motifs are T-enriched (Supplemental Figure S2C).

### Unexpected transcripts discovered in Nanopore sequencing data

**Figure 4.**
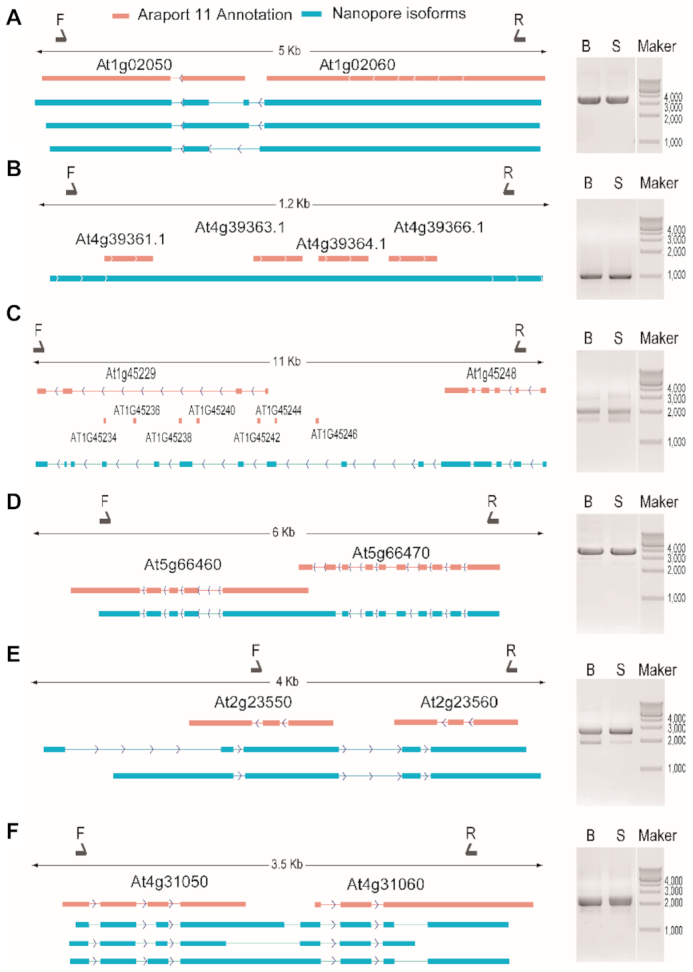
Unexpected fusion transcripts identified with Nanopore reads are confirmed with RT-PCR. (**A**) Multiple fusion transcripts derived from two discrete existing loci; (**B**) a single exon transcript covering four gene locus; (**C**) a transcript covering two distal multi-exon genes together with seven single exon genes; (**D**) fusion transcripts covering two overlapped genes with the same splicing patterns, and there is no splicing in the overlapped regions of the two covered genes; (**E**) antisense transcripts covering two adjacent genes; (**F**) transcripts with different splicing pattern covering parts of two proximal gene loci. Existing exon and intron are depicted as red thin and thin bar respectively, exon and intron derived from Nanopore reads are depicted as blue thin line and thin bar respectively. The position of forward (F) and reverse (R) position are shown in the diagram. The RT-PCR confirmation results of the fusion transcripts are shown in the right of each panel. B, floral buds; S, seedlings; Maker, the 1 kb DNA ruler.

With ONT DRS long reads and TrackCluster, six types of fusion transcripts were identified: (i) fusion transcripts covering two loci with different splicing patterns in the intergenic regions, e.g. At1g02050–At1g02060 (Figure 4A). (ii) long non-annotated transcripts covering multiple loci with no splicing events in the intergenic regions, e.g. At4g39361–At4g39364 (Figure 4B); (iii) super-long transcripts covering multiple loci, with distinct splicing patterns, e.g. At1g45248–At1g45229 (Figure 4C); (iv) transcripts covering two loci, each retaining the same splicing patterns as annotated, with an un-spliced, intact intergenic regions, e.g. At5g66460–At5g66470 (Figure 4D); (v) partial antisense transcripts covering two gene loci with the intergenic regions spliced out, e.g. At2g23550–At2g23560 (Figure 4E); (vi) transcripts covering two gene loci with different splicing patterns compared to each annotated transcript, e.g. At4g31050–At4g31060 etc. (Figure 4F). The presence of all these transcript isoforms was verified by RT-PCR (Figure 4A–F) and Chop-RT-PCR (Supplementary Figure S3).

**Figure 5.**
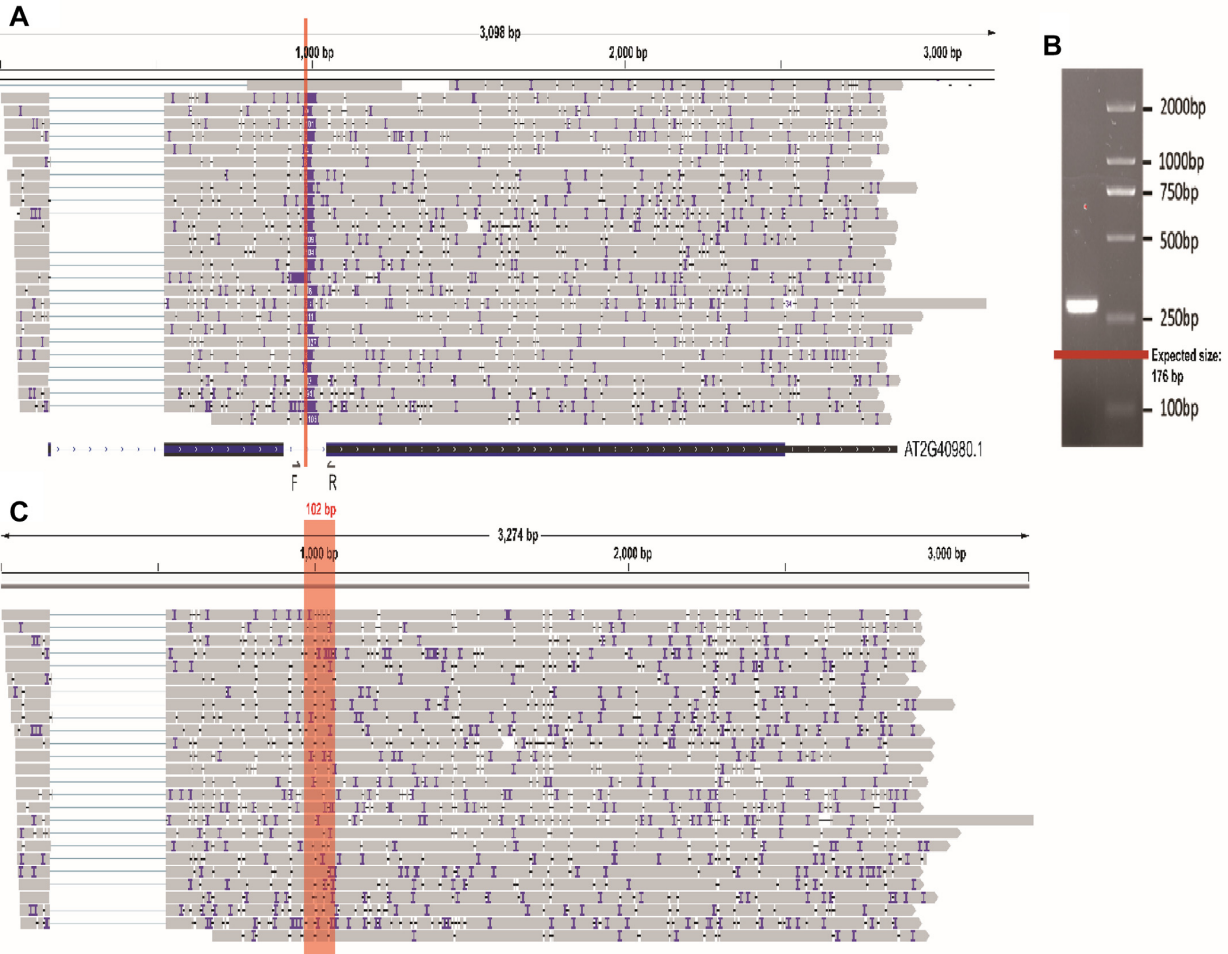
Non-annotated DNA sequence confirmed in the second intron of At2g40980. (**A**) The IGV track view of reads mapping to gene At2g40980. The unannotated sequences fall in the second intron as shown along the red line. (**B**) PCR confirmation with the primers flanking the unannotated regions, the red line indicates the expected size according to TAIR10 genome. (**C**) The IGV track view of reads with the corrected *Arabidopsis* genome. *Note*: The expected size in TAIR10 is 176 bp, while with the non-annotated DNA fragment, the expected size should be 278 bp.

Another category of novel transcripts is characterized by the inclusion of exons that are derived from the DNA sequences that are missing from the current genome assembly. For example, the gene At2g40980 has 20 reads containing a sequence of 102nt in length, which is missing from the current TAIR10 or Araport11 annotated genome in the second intron (Figure 5). The missing genomic sequence was also recovered in the ONT sequenced genome (26). We confirmed the presence of the sequence in the *A. thaliana* genome by PCR and Chop-PCR (Figure 5 and Supplementary Figure S3). The predicted size of the amplicon based on TAIR10 is 176 bp. However, the amplicon size determined in this study is ∼280 bp (Figure 5). The PCR results support the missing sequences for the second intron of At2g40980 in the current databases. Further detailed analysis is required to confirm other similar cases.

### Differentially expressed gene (DEG) and differentially expressed isoform (DEI)

**Figure 6.**
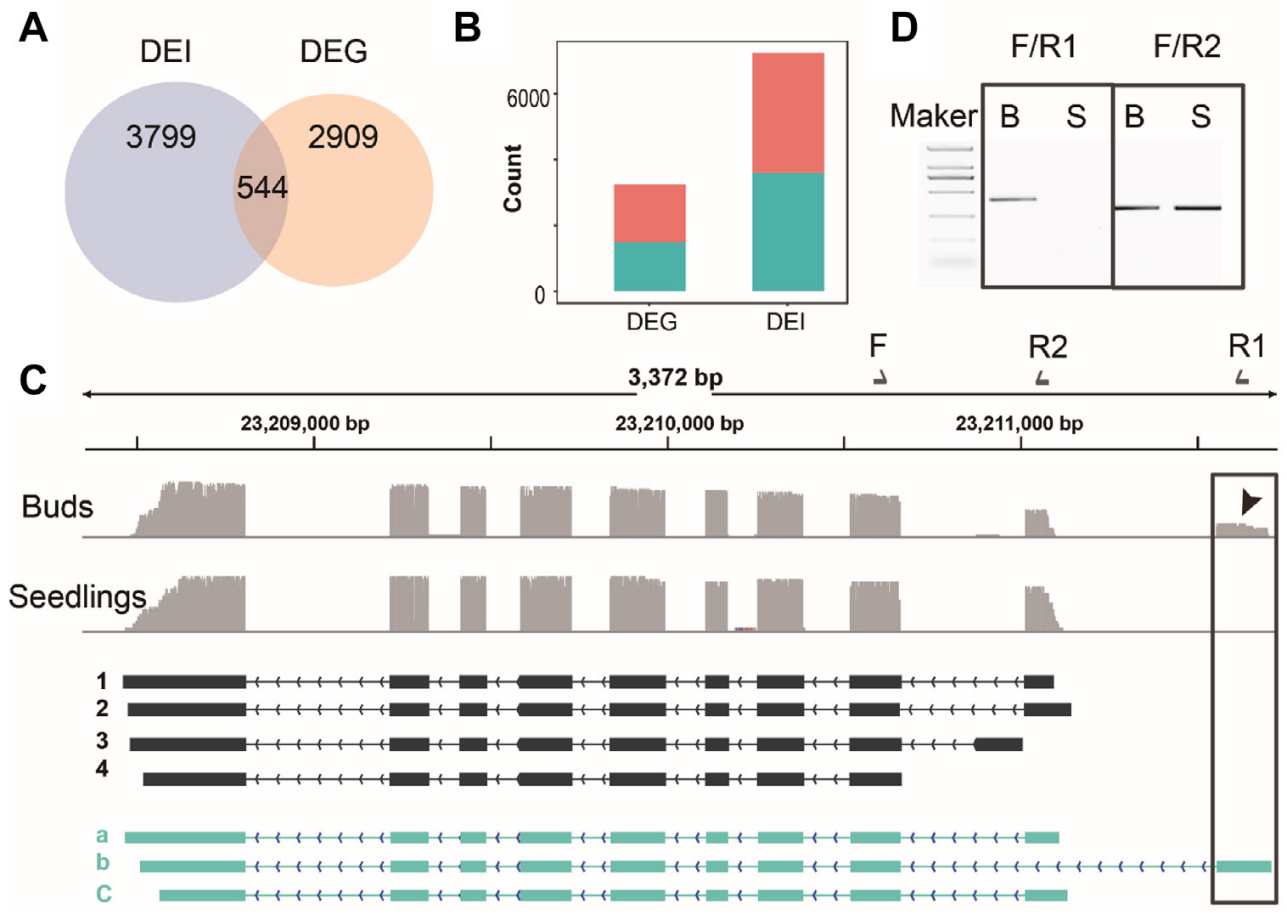
DEG and DEI between seedlings and floral buds. (**A**) Venn diagram showing the intersection of DEI and DEG, (**B**) counts of the up- and down- regulated DEG and DEI in seedling compared with floral bud, (**C**) an example of IGV track showing floral bud-specific ATI in the gene At5g57300, (**D**) experimental confirmed floral bud specific ATI events. The existing and novel isoforms are depicted in black (1–4) and green (a–c) respectively. A previously undefined ATI that is bud-specific is highlighted by a box. The primers used to validate these isoforms are labelled in the diagram. F, forward primer; R1, reverse primer 1; R2, reverse primer 2.

Using the TrackCluster algorithm with the pre-set parameter (≥5 reads support for each isoform), we identified 7111 DEIs corresponding to 4343 genes in transcripts from seedlings and floral buds (Figure 6A and B, supplementary Table S2). In addition, we identified 3453 different DEGs between the seedling and floral bud samples (Figure 6A and B). To establish relationships between DEGs and DEIs, we overlapped the DEGs and genes exhibiting the DEIs. We found that the overlapping genes accounted for only 12.5% and 15.8% of the genes having DEIs and DEGs respectively (Figure 6A). These findings indicate that expression of the tissue phenotypes is regulated at the transcript isoform level as well as the gene level. Our results demonstrate however that most genes having stage-specific DEIs may show no obvious differences at the gene level, a finding that highlights the importance of profiling gene expression at the isoform level.

The TrackCluster analysis of the DEIs successfully identified tissue specific ATI, AS, APA among others. For example, ONT DRS identified a floral bud specific ATI in At5g57300 (Figure 6C). Interestingly, the novel bud-specific isoform starts at the 3′end of the neighboring gene. However, the first exon of At5g57300 is missing. We confirmed the presence of this isoform using RT-PCR with isoform-specific primers (Figure 6D) and Chop-(RT)-PCR (Supplementary Figure S3).

### Mobile mRNAs accumulate more m5C modifications

The ONT Tombo software (27) used to detect base modifications via *de novo* model and m5C model respectively, revealed that overall RNA modifications were similar in seedlings and floral buds. Adenine modifications showed the highest accumulation at the 3′UTR near stop codon, similar to a previous report based on the meRNA-IP method (28). To demonstrate the reliability of the m5C model, we compared our data with the *A.thaliana* m5C modification datasets identified via the bisulfite sequencing method (29) or via the meRNA-IP method using anti-m5C antibody (30). This analysis demonstrates that the TOMBO m5C model is a relatively reliable algorithm for the analysis of m5C modifications of *A.thaliana* mRNAs (Supplementary Figure S4) because most of bisulfite sequencing identified genes (>75%) were covered, together with about one-third of meRNA-IP identified genes. More than 50% of the Tombo-identified m5C-containing genes were also covered by the bisulfite sequencing or the meRNA-IP methods (Supplementary Figure S4).

**Figure 7.**
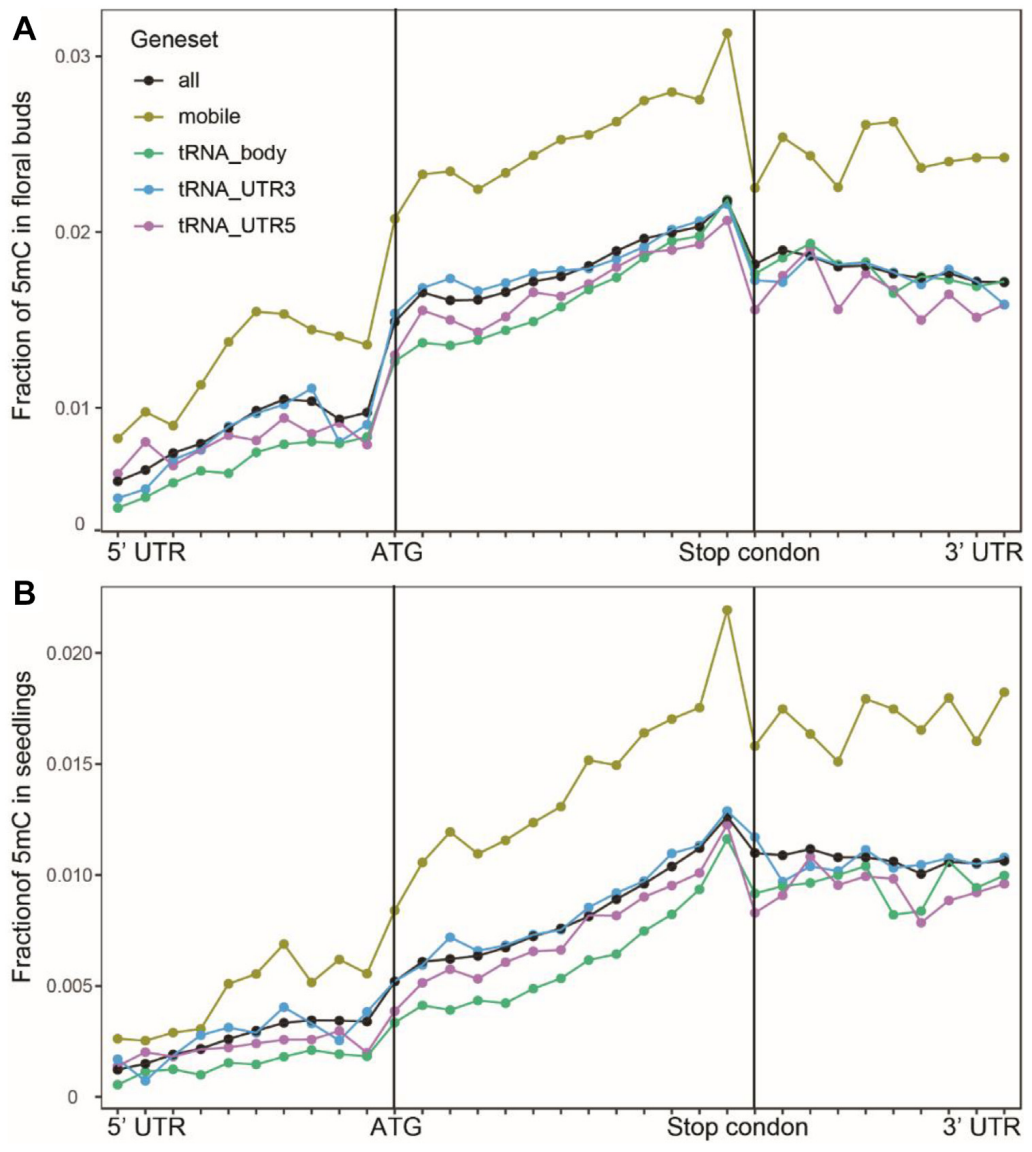
Comparison of m5C modifications between mobile mRNAs, total mRNAs and tRNA-like structure (TLS) contained mRNAs. (**A**) Comparison of m5C modification among mobile mRNAs, total mRNAs and mRNAs containing TLS in floral buds. (**B**) Comparison of m5C modification among mobile mRNAs, total mRNAs and mRNAs containing TLS in seedlings. Red: total mRNAs, light yellow: mobile mRNAs, green: TLS-containing (in gene body) mRNAs, light blue line: TLS (in 3′UTR) -containing mRNAs, purple line: TLS (in 5′UTR) -containing mRNAs.

The m5C modification in mRNAs determines the long-distance movement of mobile mRNAs (7). Using our mobile mRNA database (31), we compared m5C modifications in curated mobile mRNAs and in total mRNAs. A higher level of m5C modifications was detected in the mobile mRNA population from seedlings and floral buds than in total mRNAs (Figure 7A, B). This result supports the notion that m5C plays a key role in the long-distance movement of mobile mRNAs. The m5C modification data can also be used to investigate long-standing controversies. For example, previous studies have shown that bases in tRNAs have the highest possibility of modification (32,33) and that met-tRNA fused to cell-autonomous GUS mRNAs can promote the long-distance movement (34). In contrast, the *in silico* predicted tRNA-like structures (TLS) in endogenous mRNAs do not alone promote mRNA mobility (35). We therefore hypothesized that the key determinant for mRNA mobility is the m5C modification. Conversely, the *in silico* predicted TLS in mRNAs may not be modified by TRM4B, a homolog gene of NSUN2 in *Arabidopsis* (7,33). To test this hypothesis, we retrieved mRNAs with a predicted TLS and compared the m5C modifications between TLS-containing mRNAs and total mRNAs. No m5C enrichment was found in the TLS-containing mRNAs (Figure 7A, B). These data provide further support to the concept that the m5C modification, and not that TLS structure, determines the long-distance movement of mobile RNAs.

## DISCUSSION

ONT is a powerful technology for the generation of long reads for DNA sequencing (36). ONT DRS was recently used to measure the length of poly(A) (4) and identify a range of novel transcript isoforms including those with AS, ATI and APA, as well as fusion transcripts in the *vir-1* mutant (4,37,38). Although ONT DRS can, in theory, resolve the problems faced by Next-Generation Sequencing (NGS) (39), native RNA molecules are very easily folded into secondary structures or higher order structures such as RNA G-Quadruplex structure *in vitro* (5). These problems may prohibit the sequencing of full-length transcripts. During the initial trials with standard protocols for the libraries, we were unable to retrieve any full-length transcripts for At4g17140 (>13 000nt). However, with the improved protocol based on the observation that the lithium ion (Li^+^) can disrupt RNA-G-Quadruplexes (40), we were able to acquire 4 full-length At4g17140 transcripts. We identified putative transcripts differing from ones annotated in Araport11. This finding indicates that the improved protocol for library preparation used in the study yields better results for full-length RNA sequencing.

Recently, Parker *et al.* reported an ONT DRS analysis of *Arabidopsis*, providing novel results regarding various aspects of the transcriptome, such as, spurious antisense reads are rare or absent in Nanopore DRS; basecalling errors are non-random in Nanopore DRS. The study also pointed out that first 13 nt of the 5′ end of a transcript may not be effectively identified because of a loss of processing control by the motor protein when the end of an RNA molecule enters the pore, etc. Parker *et al.* also used the m6A mutant *vir-1* to further investigate m6A effects on gene expression and regulation as well as circadian rhythm control (4). In the present study, we used long reads derived from *Arabidopsis* wild type Col-0 seedlings and from floral buds and a recently published algorithm TrackCluster (18) to discover novel transcript isoforms. The major differences between the data reported in the present study and that published by Li *et al.* are due to our use of an improved preparation method of sequencing libraries and trans-splicing leader issues when analyzing transcriptome with TrackCluster. In the present studies, we used a modified method with an extra treatment step that involved the addition of lithium to disrupt RNA-G-Quadruplexes. Furthermore, the trans-splicing leader (SL) only occurs at the 5′ end of *Caenorhabditis elegans* RNAs and it is absent from *Arabidopsis*. Here we identify >38 500 novel transcript isoforms, revealing tissue-specific DEG and DEI forms and cryptic exons such as the one described in At2g40980, which is not annotated in the TAIR10 genome. We have also used the Tombo algorithm (ONT company provided) to identify m5C modifications in *Arabidopsis* transcripts. We demonstrate that mobile mRNAs are highly enriched in m5C modifications. Although both studies (our studies and Parker *et al.*) on *Arabidopsis* have used data generated by ONT DRS, the two studies are focused on very different aspects of mRNAs biology. A common conclusion from both studies is that ONT DRS offers significant advantages in transcriptome analysis and in RNA biology.

Transcriptome analysis is a fundamentally important holistic approach to functional genomics (41), particularly in *A. thaliana* (42). In addition, mRNA processing such as AS is attracting increasing attention because of the potential functional significance of different transcript isoforms in environmental or developmental responses (43). Araport11 is one of the most comprehensive databases for *A. thaliana* transcript isoforms (6). The data contained in Araport11 are largely derived from NGS sequencing of cDNAs. It is predicted therefore that ONT DRS will reveal novel transcript isoforms (>38 500) because this technique is able to recover intact full-length transcripts. Nearly all of the novel transcript categories were experimentally confirmed with (RT)-PCR and chop-(RT)-PCR. These observations suggest that most of our novel isoforms identified in this study exist in nature. However, we cannot rule out the possibility that some of the isoforms identified here may arise from incomplete splicing of pre-mRNAs. In contrast, PCR-amplified short reads usually cover only partial transcripts. To compare the transcriptome information obtained by NGS and ONT DRS, we used the reported transcriptome data from 14-day-old *Arabidopsis* seedlings (13) and from *Arabidopsis* floral buds at stage 8–10 (14). The two developmental stages analysed in the present study revealed few difference between the present results and the NGS data reported in the literature. Moreover, the detected Araport11 annotated transcripts and transcript isoforms reveal no obvious differences between the two sequencing platforms (Figure 1B). The correlation co-efficient of expressed genes in floral buds and in seedlings is >0.73 between ONT DRS data and NGS data, when NGS data were standardized with TPM (transcripts per million), and ONT DRS data with CPM (counts per million) (Supplementary Figure S5A). Although >50% of the ONT DRS identified DEG were confirmed in the NGS datasets (Supplementary Figure S5B), the comparison is limited by the huge differences in the two sequencing platforms.

DRS data contain base modification information. The finding that mobile mRNAs accumulate m5C modifications (Figure 7A, B, supplementary Table S3) supports the recent report that m5C facilitates mRNA long-distance movement (7). The results are further supported by bisulfite-sequenced data (29), in which 78.4% of the m5C-containing transcripts belong to mobile RNA categories based on our curated mobile mRNA database (31). Discrepancies between observations that met-tRNA fused to autonomous RNA facilitate RNA long-distance movement (34) and the finding that TLS-containing mRNAs may not be mobile mRNAs (35) can be resolved with our results, which indicate that TLS-containing mRNAs do not always show cytosine methylation. We previously sequenced 30 to 100 nt of pumpkin phloem sap RNAs, and demonstrated that the native full-length and truncated tRNA fragments in sieve elements contain m5C modified bases (44). Furthermore, TCTP1 and HSC70.1 mRNA mobility was found to be dependent on transcript cytosine methylation (7). Together, these observations establish a strong connection between m5C modifications in RNA (tRNA and mRNA) and their long distance mobilities.

## DATA AVAILABILITY

All data are available from the corresponding author upon request. The primers used in the study to confirm the observed novel isoforms can be found in Supplementary Table S4. The fastq sequences for all 4 libraries have been deposited in the GenBank database under the accession codes GSE144828.

## Supplementary Material

gkaa588_Supplemental_Files
