# Supplementary material for: New insights into Arabidopsis transcriptome complexity revealed by direct sequencing of native RNAs
Source: Nucleic Acids Res. 2020 Jul 11;48(14):7700–11. doi: 10.1093/nar/gkaa588 (PMC7430643; doi:10.1093/nar/gkaa588)
Supplement: gkaa588_Supplemental_Files [file gkaa588_supplemental_files.zip › NAR-Supplementary file.pdf]

Supplementary Information for

New insights of *Arabidopsis* transcriptome complexity revealed by direct sequencing of native RNAs

Shoudong Zhang[1,#,*], Runsheng Li[2,#], Li Zhang[1], Shengjie Chen[1], Min Xie[1], Liu Yang[1], Yiji Xia[2], Christine H. Foyer[3], Zhongying Zhao[2,*], Hon-Ming Lam[1,*]

Shoudong Zhang, Zhongying Zhao, Hon-ming Lam
Email: shoudongzhang@cuhk.edu.hk; zyzhao@hkbu.edu.hk; honming@cuhk.edu.hk

This PDF file includes:

- Figures S1 to S5
- Tables S1 to S5
- TrackCluster code and Sanger sequencing data

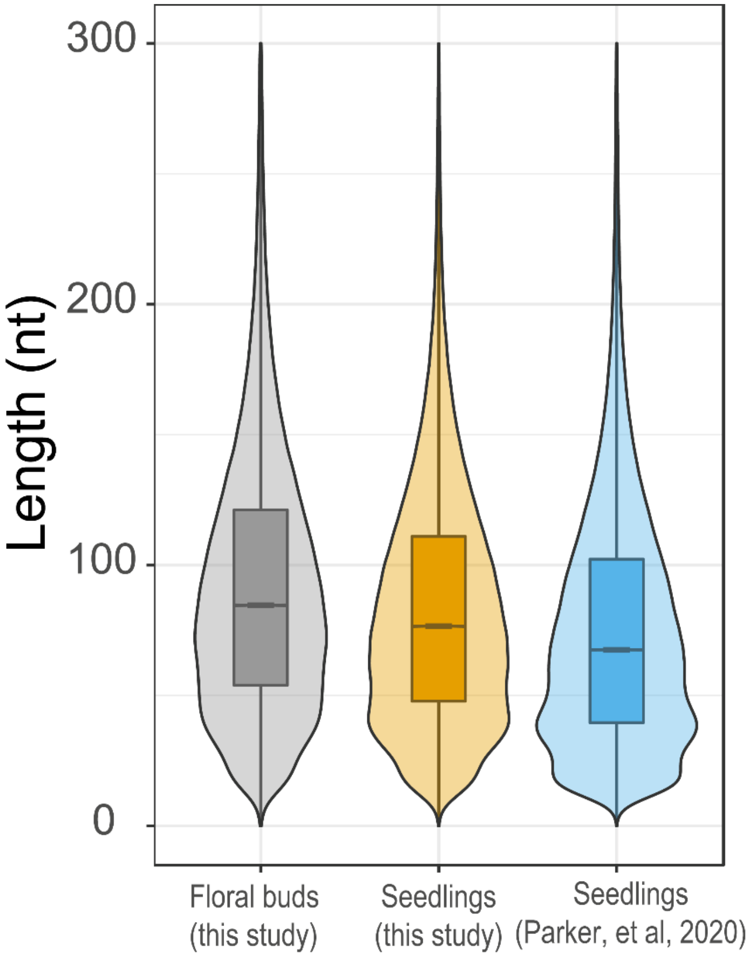

Figure S1. The Poly(A) length distribution in floral buds and in seedlings.

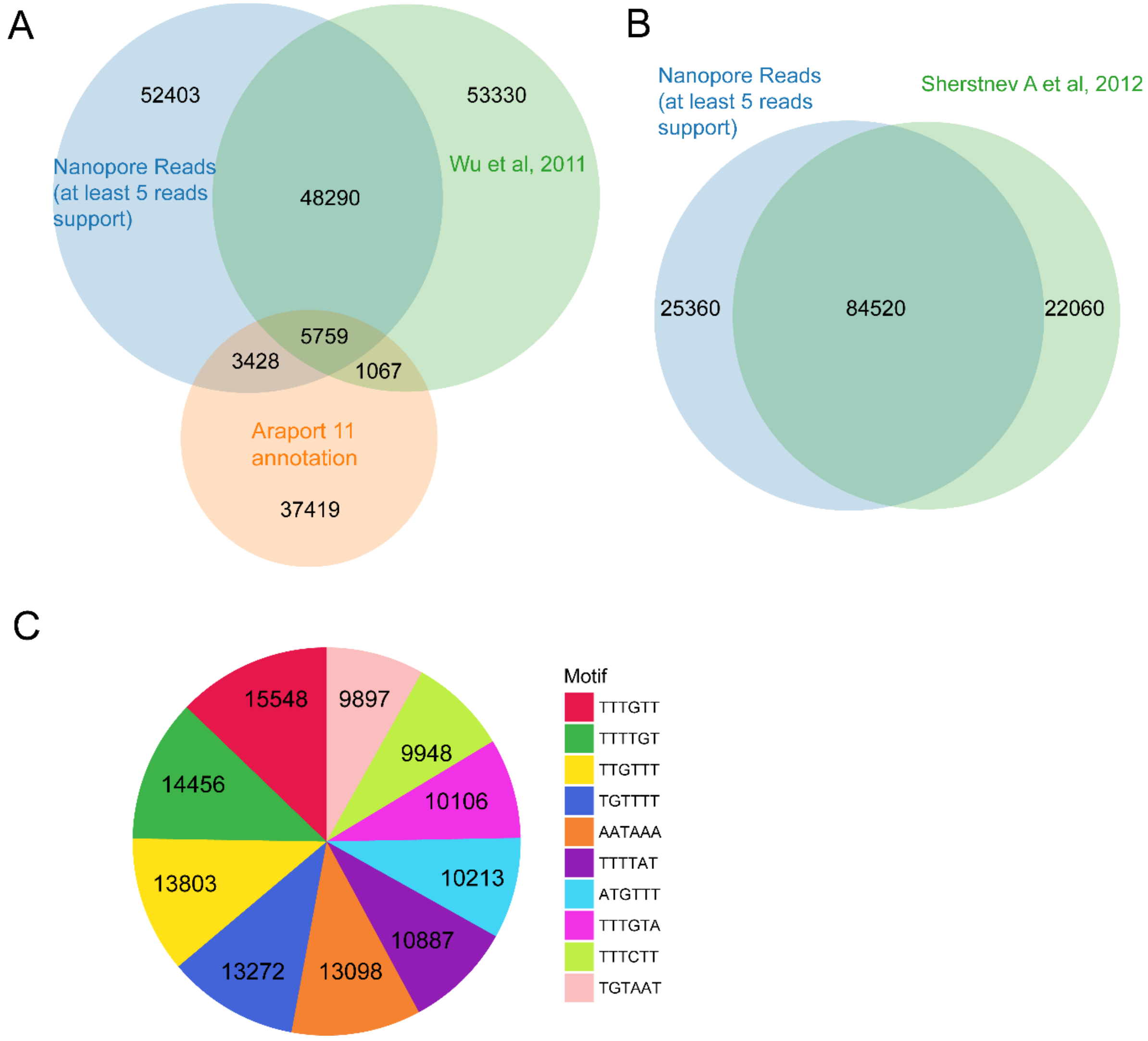

Figure S2. No. of Poly(A) sites and categories of poly(A) signal motifs. (A) Diagram showing the intersection of polyadenylation sites with at least 5 Nanopore reads support with the sites from Araport11 and from the research paper of Wu et, al. 2011. (B) Diagram showing the intersection of polyadenylation sites with at least 5 Nanopore reads support with the sites from Shestnev et al. 2012. (C) Top 10 polyadenylation signal motifs for alternative PAS identified using all the 109, 880 polyadenylation sites from (A).

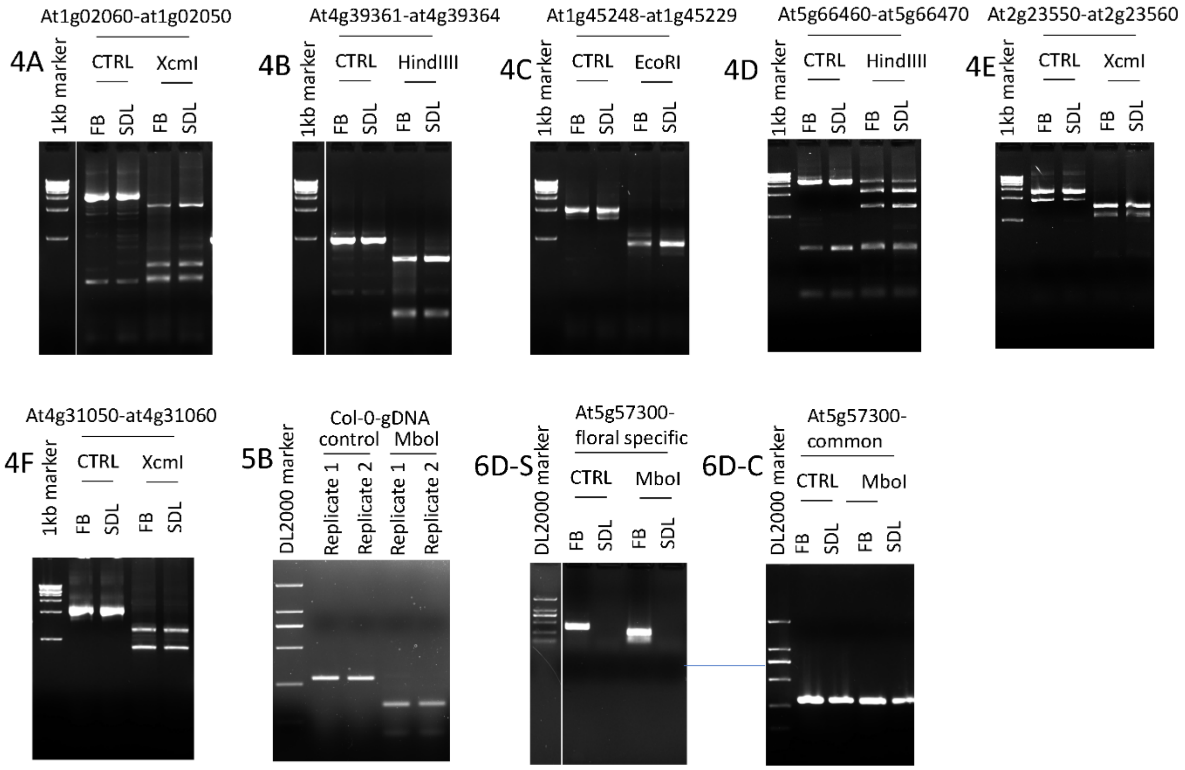

Figure S3: Chop-(RT)-PCR to confirm the fusion transcripts. 4A: At1g02060-at1g02050, expected sizes are 3452bp, after XcmI digestion, the expected sizes are 2401bp, 598bp and 453bp; 4B: at4g39361-at4g39364, expected sizes are 1016bp, after HindIII digestion, the expected sizes are 668bp, 167bp and 181bp; 4C: At1g45248-At1g45229, the expected sizes are 2203bp, after EcoRI digestion, the expected sizes are 1022bp, 929bp and 252bp; 4D: At5g66460-at5g66470, expected sizes are 3785bp, after HindIII digestion, the expected sizes are 2232bp, 1345bp and 208bp; 4E: At2g23550-at2g23560, expected sizes are 2647bp, after XcmI digestion, the expected sizes are 1513bp and 1134bp; 4F: At4g31050-at4g31060, the expected sizes are 2001bp, after XcmI digestion, the expected sizes are 1225bp and 776bp; 5B: Unannotated DNA sequence confirmation with PCR and chop-PCR. The expected size for the DNA fragment with the unannotated DNA sequence is 278bp, after MboI digestion, the expected sizes are 153bp, 67bp and 58bp. 6D-S: At5g57300-floral specific, the expected sizes are 404bp, after MboI digestion, the expected sizes are 285bp and 119bp; 6D-C: At5g57300-common, the expected sizes are 304bp, after MboI digestion, the expected sizes are 285bp and 19bp. 4A-4F corresponding to Figure 4 (A to F panels); 6D-S, Figure 6 panel D-floral specific; 6D-C, Figure 6 panel D-common fragment; FB: floral buds; SDL: seedlings; CTRL: control.

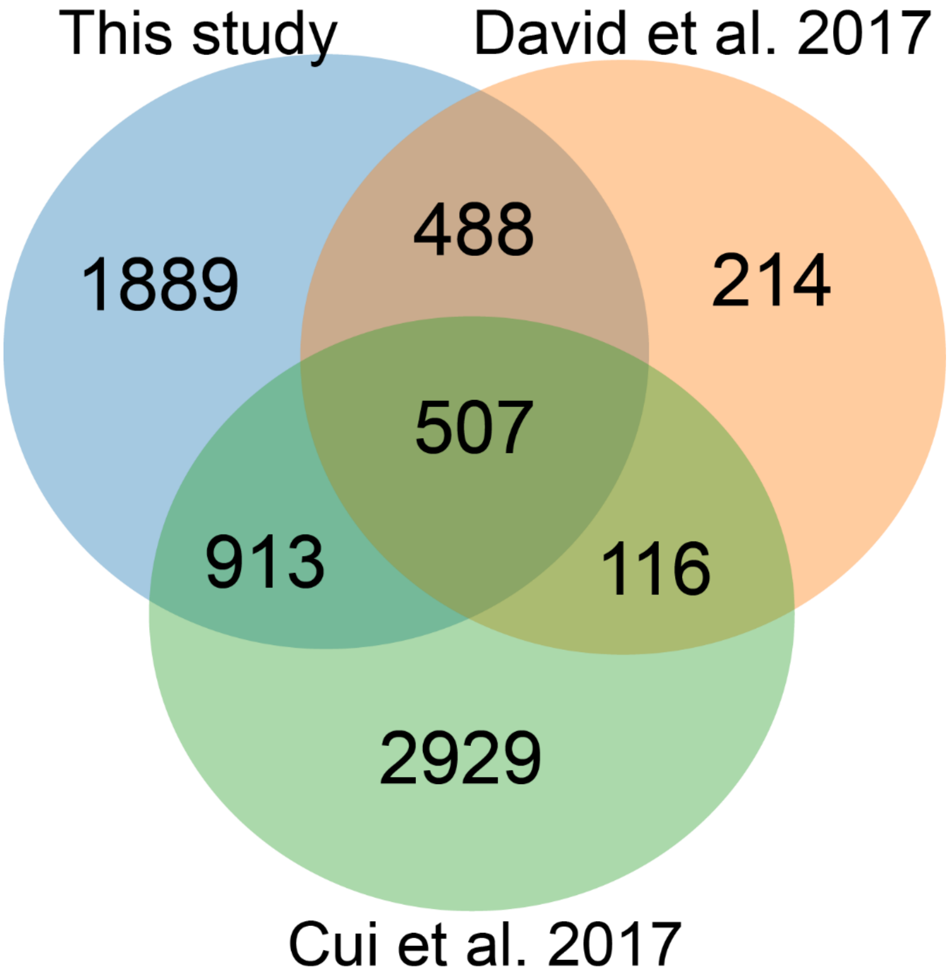

Figure S4. Venn diagram showing the intersection between the positive m5C genes in different studies. The orange cycle indicates the genes with at least one positive site in the RNA bisulfite sequencing results from David et al. (2017); while the green cycle indicates the genes with at least one positive locus in the m5C MeRNA-IP results of the wild type samples from Cui et al. 2017.

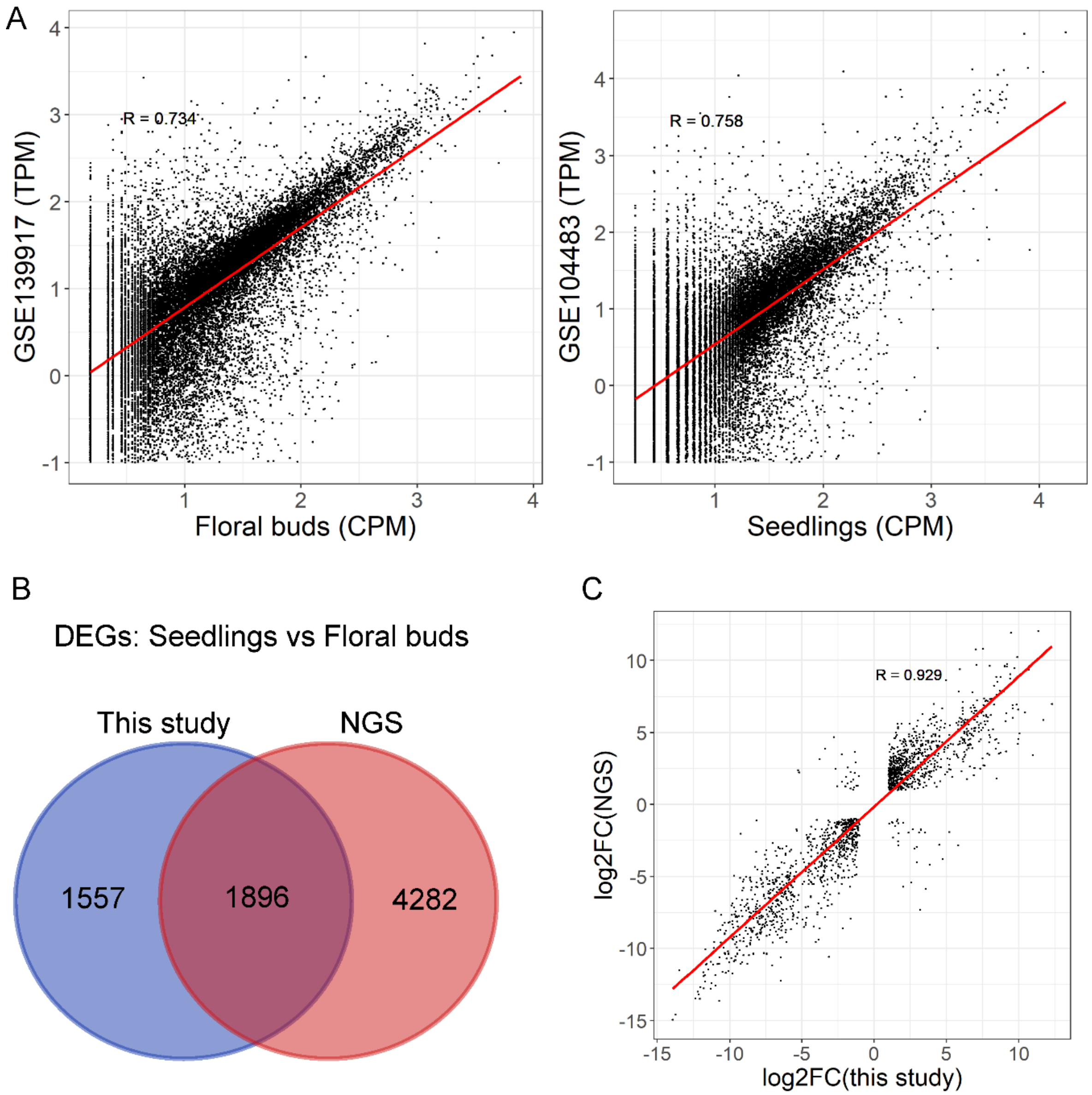

Figure S5. Comparison of NGS data and our DRS data. (A) The correlation between the average RNA expression of floral buds of our DRS reads (n=2) with NGS reads (n=4), and between the average gene expression of seedlings of our DRS reads (n=2) with NGS reads (n=3). (B) Venn diagram showing the intersection of the DEGs between two tissues identified by our DRS reads (blue) or by NGS reads (red). (C) The correlation between the fold change of the 1898 DEGs identified by both DRS and NGS reads. The details of the NGS datasets used in this study can be found in “Material and methods”. CPM, count per million. TPM, transcripts per kilobase million. DEG, differentially expressed gene.

Other Supporting Information Files

Table S1. All identified novel transcript isoforms in Arabidopsis seedlings and floral buds.
Table S1 (xlsx)
Table S2. List of DEG and DEI between floral buds and seedlings.
Table S2 (xlsx)

Table S3. Modified position of bases in transcripts with more than 90% confidence.
Table S3 (xlsx)

Table S4. Primers used in this study.

Table S4 (xlsx)

Table S5. Summary of used published data.

Table S5 (xlsx)

Supplementary data1: TackCluster code

Supplementary data2: Sanger sequencing data
